# Corrigendum: Anderson localization and Mott insulator phase in the time domain

**DOI:** 10.1038/srep46946

**Published:** 2018-02-28

**Authors:** Krzysztof Sacha

Scientific Reports 5: Article number: 1078710.1038/srep10787; published online: 06 15 2015; updated: 02 28 2018

This Article contains typographical errors. In the Results section under subheading ‘Mott insulator phase in the time domain’.

“In order to describe behaviour of the interacting many-body system we may truncate the Hilbert space to a subspace spanned by Fock states |*n*_1_, …, *n*_*s*_〉 where the occupied modes correspond to localized wave-packets *ψ*_*j*_ moving along a *s*-resonant trajectory. Then, the many-body Floquet Hamiltonian reads
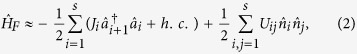
where 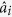 and 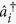 are bosonic anihilation and creation operators and 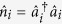. The coefficients 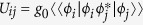 describe interactions between particles that ocupy the same mode (for *i* = *j*) and between particles in different modes (*i* ≠ *j*).”

should read:

“In order to describe behaviour of the interacting many-body system we may truncate the Hilbert space to a subspace spanned by Fock states |*n*_1_, …, *n*_*s*_〉 where the occupied modes correspond to localized wave-packets 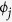 moving along a *s*-resonant trajectory. Then, the many-body Floquet Hamiltonian reads
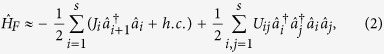
where 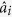 and 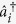 are bosonic anihilation and creation operators. The coefficients 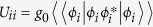 describe interactions between particles that occupy the same mode (for *i* = *j*) and 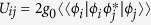 between particles in different modes (*i* ≠ *j*).”

In the Legend of Figure 2,

“Proper superpositions of the eigenstates allows one to extract 4 individual wave-packets, *ψ*_*j*_, that are numbered in (**a**) and (**b**).”

should read:

“Proper superpositions of the eigenstates allows one to extract 4 individual wave-packets, 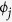, that are numbered in (**a**) and (**b**).”

In the Legend of Figure 3,

“The coefficients *α*_*n*_, in *H*′, are chosen so that the set of 〈〈*ϕ*_*j*_|*H*′|*ϕ*_*j*_〉〉 reproduces a chosen set of numbers *E*_*j*_, where *ψ*_*j*_’s are the wave-packets described in Fig. 2.”

should read:

“The coefficients *α*_*n*_, in *H*′, are chosen so that the set of 〈〈*ϕ*_*j*_|*H*′|*ϕ*_*j*_〉〉 reproduces a chosen set of numbers *E*_*j*_, where *ϕ*_*j*_’s are the wave-packets described in Fig. 2.”

and

“The wave-packets *ψ*_*j*_ arrive at a given position *z* in equidistant intervals in time, thus, the AL length in time is *l*_*t*_ = *lT*.”

should read:

“The wave-packets 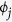 arrive at a given position *z* in equidistant intervals in time, thus, the AL length in time is *l*_*t*_ = *lT*.”

